# Public attitude toward Covid‐19 vaccination: The influence of education, partisanship, biological literacy, and coronavirus understanding

**DOI:** 10.1096/fj.202200730

**Published:** 2022-06-03

**Authors:** Jon D. Miller, Mark S. Ackerman, Belén Laspra, Carmelo Polino, Jordan S. Huffaker

**Affiliations:** ^1^ Institute for Social Research University of Michigan Ann Arbor Michigan USA; ^2^ School of Information and College of Engineering University of Michigan Ann Arbor Michigan USA; ^3^ Department of Philosophy University of Oviedo Asturias Spain; ^4^ Centro Redes Buenos Aires Argentina; ^5^ College of Engineering University of Michigan Ann Arbor Michigan USA

## Abstract

The Covid‐19 pandemic posed new issues about vaccination and contagious diseases that had not been the focus of public policy debate in the United States since the tuberculosis pandemic of the late 19th century and the early 20th century. Using a national address‐based probability sample of American adults in 2020 and a structural equation model, this analysis seeks to understand the role of education, age, gender, race, education, partisanship, religious fundamentalism, biological literacy, and understanding of the coronavirus to predict individual intention concerning taking the Covid‐19 vaccine. Given the substantial changes in the United States since the tuberculosis pandemic, it is important to understand the factors that drive acceptance and hesitancy about Covid‐19 vaccination. We find that education, biological literacy, and understanding of the coronavirus were strong positive predictors of willingness to be vaccinated and religious fundamentalism and conservative partisanship were strong negative predictors of intent to vaccinate. These results should be encouraging to the scientific community.

**Abbreviations**
ACM: Association for Computing Machinery
CHI: Computer‐Human Interaction
CFA: confirmatory factor analysis
ICPSR: Inter‐university Consortium for Political Research
LISREL: linear structural relations
SEM: structural equation model
TE: total effect

## 1 INTRODUCTION

The Covid‐19 pandemic was the most serious public health crisis in the lifetime of most American adults. Although effective vaccines were developed and made available about 12 months after the onset of the pandemic, a significant portion of American adults declined or resisted vaccination. It is important to understand the factors that are associated with vaccine acceptance, resistance, or hesitancy.^1^

The Covid‐19 pandemic differed in several ways from previous pandemics. In the 18th and 19th centuries, tuberculosis killed approximately one in seven adults and the cause of the disease was not known. Early in the 20th century, scientists discovered that tuberculosis was caused by a bacterium, but it was not until the development of effective antibiotics during and after the Second World War that widespread treatment became possible. In recent decades, a new antibiotic‐resistant strain of tuberculosis developed and the World Health Organization had made its control one of their top priorities. For most of the years prior to the development of effective pharmaceutical treatments, tuberculosis patients were sent to sanatoriums, often against their will, to prevent the spread of tuberculosis.

The polio pandemic in the 1940’s and 1950’s in the United States followed a similar pattern. The cause of polio was unknown for several years. President Franklin Roosevelt had polio as a young man and was crippled for the remainder of his life. President Roosevelt and other concerned citizens and leaders created a national organization – the March of Dimes – to collect small gifts from children and adults to be used to find the cause and a cure of polio. Given this history of national concern and the unknown cause of the disease, the discovery of a vaccine by Jonas Salk in 1955 was a source of national relief. President Eisenhower and Elvis Pressley were vaccinated publicly and there was a strong demand for polio vaccinations by adults for themselves and for their children. There was no partisan resistance to the polio vaccine.^2^

In the context of the previous experience of Americans with contagious and deadly diseases, the partisan polarization of attitudes toward Covid‐19 and Covid‐19 vaccinations is unusual and requires some explanation. The purpose of this analysis is to utilize a pair of national surveys of a probability‐sample of American adults to identify, measure, and analyze the factors associated with a positive intent to be vaccinated against Covid‐19 and a hesitancy toward or rejection of a Covid‐19 vaccination.

## 2 MATERIALS AND METHODS

### 2.1 Data

This analysis will utilize data from a national two‐wave probability sample of American adults selected and administered by Ameri‐Speak, a service of the National Opinion Research Center. An initial survey was collected in February and March of 2020, near the onset of the Covid‐19 pandemic. A second wave was collected from the same respondents in November and December of 2020 – a year into the pandemic. A total of 3141 completions were obtained in the first wave and 2737 of the same respondents were obtained in the second cycle – a retention rate of 87.1%. Respondents in both waves were offered the choice of an online survey or a telephone survey and were free to select the method most comfortable to each individual. The data from these surveys have been deposited in the Inter‐university Consortium for Political and Social Research (ICPSR) and are being processed for public use.

### 2.2 Factors associated with Covid‐19 vaccine acceptance or hesitancy

Our measure of each respondent's attitude toward Covid‐19 vaccination is based on a question asking each respondent:

If a tested and effective vaccine were to become available, would you:

-
  - definitely take it
  - probably take it
  - take it only if required for my job or by law
  - refuse to take it
  - not sure what I would do

These responses were recoded into a five‐category ordinal variable: (1) Refuse, (2) Only if required, (3) Not sure, (4) Probably, or (5) Definitely. Using a national address‐based probability sample, 49% of American adults indicated in November and December 2020 that they would definitely or probably seek a Covid‐19 vaccination as soon as a safe and effective vaccine became available (see Table 1). This ordinal measure has been used widely in the Covid‐19 literature^1^ and is often referred to a *vaccination intent*.

**TABLE 1:** Attitude toward Covid‐19 vaccination, November–December, 2020

	Attitude toward Covid‐19 vaccination					*N*
	Refuse	If required	Not sure	Probably	Definitely	
All U.S. adults	17%	12%	22%	24%	25%	2737
Respondent age *γ* = 0.25						
18–29 years	22%	16%	22%	18%	22%	562
30–39 years	23	18	20	23	16	474
40–49 years	20	14	25	25	16	433
50–59 years	17	13	26	24	21	455
60–69 years	14	4	20	25	35	453
70 and more years	3	4	14	35	44	360
Race *γ* = −0.26						
Other	17	12	20	25	26	2410
African‐American	20	15	36	18	11	326
Gender *γ* = −0.29						
Males	14	10	16	28	32	1321
Females	21	14	26	21	18	1415

Note

Gamma is a proportional‐reduction‐of‐error statistic (see Ref. [8]).

In the summer of 2020, two non‐probability surveys of American adults reported substantially higher levels of likely public acceptance. Using a set of volunteer participants (adjusted to reflect a quota sample) in each of 19 countries,^3^ estimated that 75% of Americans would take the Covid‐19 vaccine if it were available. In a study of volunteers recruited through social media in four countries,^4^ reported that 63% of U.S. respondents were definitely or probably willing to take the Covid‐19 vaccine. These over‐estimates illustrate the value of probability sampling and the hazards of volunteer samples.

Even though 59% of European respondents did not expect the availability of a vaccine by the end of the year, 74% of Europeans indicated in September 2020 that they would take the Covid‐19 vaccination if it were available. Significantly lower values were noted for France, Russia, Hungary, and Poland.^5^ In the months of October, November and December, the European data showed a decrease in confidence in the vaccine and an increase in reluctance to be vaccinated.

It is useful to recall that the Pfizer and Moderna Covid‐19 vaccines were developed during 2020 and that clinical trials were completed just before the end of calendar 2020. The Food and Drug Administration gave the two vaccines an Emergency Use Authorization in the U.S. in mid‐December 2020. The second wave of our 2020 survey was conducted in November and December of 2020 with a small number of completions collected in January and early February 2021. Our question about the willingness of respondents to take a Covid‐19 vaccine asked about respondents’ willingness to take a “tested and effective vaccine” when it became available without specifying a date. This measure is a good indicator of the predisposition to take a Covid‐19 vaccine apart from any subsequent discussions about the efficacy of any specific vaccine.

## 3 RESULTS

### 3.1 Three basic demographic variables

We begin with a review of three exogenous variables acquired at birth: age, race, and gender.

Older respondents were significantly more likely to express a definite or probable willingness to be vaccinated for Covid‐19 than younger adults (see Table 1). An ordinal measure of respondent age accounted for 25% of the mutual dependence (variance) in this relationship.^a^ For several decades, older adults were advised to take an annual vaccination against influenza and about 75 000 individuals die from influenza each year in the United States. There was wide media coverage of the higher Covid‐19 death rate among older adults in both nursing homes and in regular housing arrangements. As the Covid‐19 pandemic progressed into 2021, public health messages urged all adults to be vaccinated against Covid‐19 regardless of age. Our data suggest that younger adults were less predisposed to get a Covid‐19 vaccination, perhaps reflecting the years of messages that influenza vaccination was unnecessary for younger adults. This pattern is consistent with a parallel study of older U.S. adults who were enrolled in a clinical trial related to heart health.^6^

African‐Americans were less receptive to the prospect of getting a Covid‐19 vaccination than other adults. Race accounts for nearly 30% of the variation in vaccination attitude (see Table 1). Some of this reluctance undoubtedly reflects the history of abuse in the Tuskegee syphilis experiments in the 1930’s and its echoes in later years.^7^

Male respondents were more receptive to getting a Covid‐19 vaccine than women (see Table 1). The *γ* for the relationship between gender and vaccination attitude is −0.29, indicating that gender accounts for nearly 30% of the variation in vaccination attitude.

Each of these three exogenous variables account for approximately a third of the variation in attitude toward vaccination in these bivariate cross‐tabulations. In life, these variables exist simultaneously in each individual. After a review of the major factors related to Covid‐19 vaccination attitude, we will place all of the independent variables in a structural equation model (SEM) to determine the relative influence of each variable, taking into account the chronological and logical relationships among the predictor variables.

Data about European countries shows that a higher proportion of men were willing to get vaccinated than women, and it is largest among men above the age of 55. Males who were unwilling to get vaccinated tended to be younger with the largest share of 12% among the 18–24‐year old.^9^

### 3.2 Education, biological literacy, and coronavirus understanding

A second important set of predictors of a predisposition in favor of vaccination for Covid‐19 involves each respondent's education, general biological literacy, and level of understanding of viruses generally and the coronavirus specifically. There is a large literature demonstrating that when complex science policy issues become the subject of politicized debate, the level of education and understanding become important tools to help a citizen make sense of discussions and debates involving those issues.^10^, ^11^, ^12^, ^13^

Our 2020 surveys found that the level of educational attainment was positively related to a receptive attitude toward vaccination for Covid‐19 (see Table 2). Sixty‐one percent of adults with a baccalaureate reported that they would definitely or probably get a Covid‐19 vaccination when it was available and more than 70% of adults with a graduate or professional degree made a similar report. In contrast, 39% of high school graduates were predisposed to get a Covid‐19 vaccination and only 31% of adults who did not complete high school were likely to get a Covid‐19 vaccination. Educational attainment accounted for 27% of the variation in vaccination attitude.

**TABLE 2:** Attitude toward Covid‐19 vaccination, November‐December, 2020

	Attitude toward Covid‐19 vaccination					*N*
	Refuse	If required	Not sure	Probably	Definitely	
All U.S. adults	17%	12%	22%	24%	25%	2737
Respondent education *γ* = 0.27						
Less than high school	16%	18%	35%	19%	12%	155
High school grad / GED	22	13	26	20	19	1340
Associate degree	19	12	21	27	21	258
Baccalaureate	14	9	16	28	33	566
Master's degree	9	10	9	35	37	317
Doctorate/professional	5	8	13	22	52	101
Number of college science courses *γ* = 0.27						
None	21	13	27	21	18	1481
1–3 courses	16	12	18	28	26	600
4 or more courses	11	10	14	27	38	657
Score on biological literacy index *γ* = 0.43						
0–69 not literate	20	13	24	23	21	2220
70–100 literate	7	8	12	30	43	517
Score on understanding of coronavirus index *γ* = 0.54						
0–69	28	15	27	18	13	1407
70–100	6	9	17	31	37	1331

Note

Gamma is a proportional‐reduction‐of‐error statistic (see Ref. [8]).

One of the unique features of American higher education is the requirement that all baccalaureate graduates complete at least a year of undergraduate science courses regardless of their major. This minimum requirement of a year of science courses has produced a relatively high level of scientific literacy among U.S. adults^14^, ^15^, ^16^, ^17^ and has produced a mass market for new scientific and medical products. The data from our 2020 study demonstrates the power of college science courses. Fifty‐four percent of American adults who completed one to three college science courses – the general education requirement in most universities – were definitely or probably willing to take a Covid‐19 vaccine as soon as it was available, and 65% of adults with four or more college science courses were positively disposed to take a Covid‐19 vaccination (see Table 2). This relationship accounted for 27% of the variation in vaccination attitude.

A widely used indicator of adult biological literacy^11^, ^12^ was included in our 2020 U.S. survey. Nineteen percent of American adults qualified as literate on the Biological Literacy Scale (see Table 2) and 73% of biologically literate adults indicated that they would definitely or probably take a Covid‐19 vaccination when it was available. Only 44% of adults who did not qualify as biologically literate were inclined to be vaccinated against Covid‐19. Biological literacy accounted for 43% of the variation in vaccination attitude.

The biological literacy measure is a good indicator of a broad understanding of important biology constructs – cells, stem cells, bacteria, viruses, DNA – but it does not include any items specifically focused on the coronavirus or on the nature of a pandemic. To provide a more precise measure of respondent understanding of the coronavirus, our 2020 fall survey included a set of six items that a confirmatory factor analysis (CFA) found to constitute a unidimensional scale. The CFA factor score was converted into a 0‐to‐100 index. A dichotomous version of this index (scores of 70 or more classified as high, scores <70 classified as low) indicates that it is a very strong predictor of a positive predisposition toward vaccination against the coronavirus, accounting for 54% of the variation in vaccination attitude (see Table 2). Sixty‐eight percent of adults scoring 70 or higher on the index indicated that they would definitely or probably take a Covid‐19 vaccination as soon as it became available.

The increase in the gamma for these four relationships illustrates the predictive power of more focused indices over broader general indices. The level of educational attainment is a broad measure that cuts across all disciplines and career preparations. One individual may have a baccalaureate in history and another respondent may have a baccalaureate in cell biology, and both individuals may have had a minimum of three college science courses, but the biology major may have had 10 or 15 college science courses. Some of this variation is captured in our measure of college science courses, but this is also a relatively broad measure. An individual who earned a baccalaureate in engineering may have taken science courses in physics and geology and a student in biology may have taken a larger number of courses in cell biology, genetics, and biochemistry. We would not expect that the background knowledge of science related to the coronavirus would be the same.

Our measure of biological literacy begins to narrow the focus to an understanding of biology, which produces a higher gamma and a stronger predictor of vaccination attitude. Our index of coronavirus understanding is focused on the science of greatest relevance to formulating an attitude toward vaccination against Covid‐19 and it has the highest gamma of these four indicators. Our concluding SEM will assess the relative predictive power of each of our independent variables in an appropriate chronological and logical context.

### 3.3 Political partisanship and religious beliefs

In the first decades of the 21st century, many public policy issues have become politicized and the current partisan division in the United States is closely related to religious beliefs.^18^, ^19^ It is instructive to contrast the apolitical public reaction to the polio pandemic of the 1950’s with deeply polarized partisanship associated with the Covid‐19 pandemic.^2^ Our 2020 U.S. study included an ordinal measure of ideological partisanship and an ordinal index of religious fundamentalism (see Table 3). Both measures have been used in numerous published studies predicting both political behaviors and attitudes toward science and technology.^20^, ^21^, ^22^, ^23^, ^24^, ^25^, ^26^

**TABLE 3:** Attitude toward Covid‐19 vaccination, November‐December, 2020

	Attitude toward Covid‐19 vaccination									*N*
	Refuse		If required		Not sure		Probably		Definitely	
All U.S. adults	17%		12%		22%		24%		25%	2737
Ideological partisanship *γ* = 0.18										
Conservative republican	24%		10%		18%		24%		24%	566
Moderate republican	23		11		19		24		23	218
NP conservative	20		15		24		21		20	221
NP low political interest	25		20		27		17		11	356
NP high political interest	13		13		28		26		20	363
NP liberal	12		14		17		27		30	147
Moderate democrat	14		13		25		23		25	444
Liberal democrat	6		4		14		32		44	418
Score on index of religious fundamentalism *γ* = −0.19										
0 Secular	9	9		15		29		38		429
1–2	14	14		20		22		30		746
3–4	16	15		25		25		19		664
5–6	24	9		26		23		18		604
7–10 Fundamentalist	25	12		20		24		19		294

Note

Gamma is a proportional‐reduction‐of‐error statistic (see Ref. [8]).

Abbreviation: NP, nonpartisan.

Our measure of ideological partisanship is an extension and modification of an ordinal scale first used in the landmark *The American Voter*.^27^ The original seven‐point ordinal scale was anchored by Strong Republicans on one end and Strong Democrats on the other end. The middle of the scale was labeled independents or non‐partisans.^28^ documented the non‐ideological nature of the political attitudes of most American adults and^29^ characterized the political system as a four‐party structure reflecting regional and racial divides. By the beginning of the 21st century, the partisan division was ideological with a high level of issue alignment^20^ and with an increasing level of hostility toward the opposing party.^21^, ^22^, ^23^, ^26^ Reflecting this new reality, we adjust the ordinal measure of partisanship to range from Conservative Republicans to Liberal Democrats and rename the scale as *ideological partisanship*.

Using this scale, we find a modest, but significant, relationship between ideological partisanship and Covid‐19 vaccination attitude (see Table 3). The *γ* for this relationship is 0.18, meaning that 18% of the variation in vaccination attitudes can be accounted for by ideological partisanship. Seventy‐six percent of Liberal Democrats were likely to get a Covid‐19 vaccination compared to 48% of Conservative Republicans. Although a great deal of the political science literature has characterized the non‐ideological nonpartisans in the center of the scale as uninterested in politics or public policy issues, we find that the 26% of American adults in this center group as nearly equally divided between individuals with a low level of political interest and engagement and an equal‐sized group of adults who eschew partisanship *per se* but report a high level of political and issue interest. We differentiate these two groups in our interval scale of ideological partisanship, and – as expected – 46% of nonpartisan adults with a high degree of political interest were inclined to get a Covid‐19 vaccination compared to only 28% of nonpartisans with less political interest.

Although there has been a segment of American adults with conservative religious beliefs from the founding of the colonies, their involvement with partisan politics was limited and seldom done in the name of their church or denomination. The struggles over slavery and the Civil War caused some denominations to split – the Baptists in the South formed the Southern Baptist Convention. Although the division over race and slavery continued into the 20th century, it was not until the Baptist minister Jerry Falwell formed the Moral Majority movement in the 1970’s which led to an open linkage between religious fundamentalism and the Republican Party. This linkage – under various names – continues.^18^, ^19^

Our 2020 study included a short set of items^b^ that capture Protestant fundamentalism. A CFA was used to verify that the five items form a single attitudinal dimension and the factor scores from the CFA were used to construct a 0‐to‐10 scale. For presentation purposes, the 0‐to‐10 scale has been reduced to five ordinal categories (see Table 3). Forty‐three percent of the most fundamentalist respondents indicated that they were likely to take a Covid‐19 vaccination compared to two‐thirds of the most secular respondents in our 2020 study. The *γ* for this relationship was −0.19. This differential reflects a combination of reservations about modern science and a strong attachment to President Trump and the Republican Party.

### 3.4 Personal impact of Covid‐19, minor children, and online navigation skills

Another set of variables that have been found to be influential in earlier attitude studies include the experience with Covid‐19 in terms of individual health and the health experiences of family and friends; the presence of minor children in the respondent's home; and the respondent's level of navigation skills on the Internet and wireless devices. Although we do not expect these variables to have a dominant influence on vaccination attitudes, earlier studies have found that they often have smaller – but significant – influence on attitudes and behaviors.^13^

Our measure of personal health experiences related to Covid‐19 is a typology based on responses to several questions about the health experiences of the respondent, his or her spouse or partner, other family members, and friends and co‐workers. Using these responses, we constructed a typology that ranges from no positive test results for the respondent to a Covid‐related death of a member of the respondent's household (see Table 4). Approximately half of respondents who experienced a Covid‐related death of a friend or family member indicated a willingness to be vaccinated against Covid‐19 compared to 46% of individuals without a positive Covid test result personally or among friends or family. This relationship accounted for only 6% of the variation in vaccination attitudes.

**TABLE 4:** Attitude toward Covid‐19 vaccination, November–December, 2020

	Attitude toward Covid‐19 vaccination					*N*
	Refuse	If required	Not sure	Probably	Definitely	
All U.S. adults	17%	12%	22%	24%	25%	2737
Personal health impact of Covid‐19 *γ* = 0.06						
No positive tests (PT)	20%	13%	25%	21%	21%	809
PT outside household	16	12	20	26	26	1192
PT in household	20	14	34	20	12	98
Friend died from Covid	14	10	22	25	29	429
Member of household died from Covid	20	15	19	21	25	207
Minor children at home *γ* = −0.23						
No minor children	16	11	21	25	27	2034
Minor children at home	22	16	25	21	16	704
Internet navigation skills index *γ* = 0.08						
0 Low	18	15	27	21	19	343
1	19	17	24	22	18	376
2	17	11	21	24	27	501
3	17	8	24	26	25	575
4	19	13	16	26	26	450
5	16	11	18	24	31	271
6 High	14	12	20	24	30	217

Note

Gamma is a proportional‐reduction‐of‐error statistic (see Ref. [8]).

We included the presence of minor children in the home because earlier studies found that the parents of minor children were more likely to stop smoking and to engage with science‐related projects and conversations with their children. Our results indicate that parents with minor children at home were less likely to plan to obtain a Covid‐19 vaccination than adults without minor children at home (see Table 4). The *γ* for this relationship was −0.23.

Finally, earlier studies have found that the level of online navigation skills predicted both the frequency of information seeking about health or science topics that may, in turn, influence attitude formation. The relevant literature indicates that online navigation skills are generalizable across substantive domains. Individuals who have extensive experience seeking online information about current news, the weather, or any topic of personal interest will be able to utilize those navigation skills to seek information in other domains that become more salient or urgent.^30^ In our 2020 study, we utilized a CFA to identify a set of 10 items that form a single dimension measuring Internet or online usage. Our results suggest that higher levels of online navigation skills are positively related to an intent to obtain a Covid‐19 vaccination, but this relationship accounts for only 8% of the variation in vaccination attitude (see Table 4).

### 3.5 Trust in information sources and frequency of Covid information acquisition

Finally, the literature indicates that trust in information sources is often related to the selection and use of health and science information.^31^ Our 2020 study asked respondents to indicate the level of trust they would have in Covid‐19 information from 22 separate sources using a zero‐to‐10 scale, with 10 signifying the highest level of trust for Covid‐related information. We have clustered those responses into three major classifications for the purpose of this analysis.

First, we examine the level of trust in broadcast news from major network, local stations, and selected cable news providers. There is a large literature concerning public trust in broadcast news, but some recent indicators suggest a declining level of public trust in broadcast news.^32^, ^33^ Using a clustered set of ordinal categories to summarize the zero‐to‐10 scales collected in our 2020 surveys, we find a relatively high level of public trust in broadcast news (see Table 5). Given the convergence of media sources and consumer devices, we recognize that many respondents may watch local or network newscasts on an online site, but it appears that respondents are able to differentiate between broadcast news sources regardless of the device on which they view the show. The gamma for trust in Covid‐19 information from broadcast news sources was 0.40, which accounts for 40% of the variation in this bivariate relationship. A recent study,^34^ found the users of traditional news sources – local and network news, newspapers, radio were more likely to expect to become vaccinated.

**TABLE 5:** Attitude toward Covid‐19 vaccination, November–December, 2020

	Attitude toward Covid‐19 vaccination					*N*
	Refuse	If required	Not sure	Probably	Definitely	
All U.S. adults	17%	12%	22%	24%	25%	2737
Trust in broadcast news about Covid‐19 *γ* = 0.40						
−5 to −4	46%	12%	29%	9%	5%	226
−3 to −2	29	16	22	17	15	384
−1 to +1	18	14	26	24	19	1023
+2 to +3	9	9	18	32	34	772
+4 to +5	4	8	15	27	47	332
Trust in experts about Covid‐19 *γ* = 0.43						
−5 to −4	42	17	21	11	10	132
−3 to −2	41	12	22	19	6	228
−1 to +1	22	16	31	16	15	805
+2 to +3	13	11	19	30	27	909
+4 to +5	5	7	14	31	44	662
Trust in social media sources about Covid‐19 *γ* = 0.15						
−5 to −4	30	14	24	17	16	387
−3 to −2	20	13	17	24	28	773
−1 to +1	14	11	24	27	25	1405
+2 to +3	10	12	19	25	35	151
+4 to +5	4	13	39	17	26	23
Frequency of information acquisition about Covid‐19 *γ* = 0.18						
0–39 times/year	23	18	28	19	13	531
40–103 times/year	21	11	24	24	21	560
104–175 times/year	18	10	18	25	30	550
176–249 times/year	13	10	19	25	33	546
250–365 times/year	13	11	20	29	27	552

Note

Gamma is a proportional‐reduction‐of‐error statistic (see Ref. [8]).

Second, we examine the trust in local and national expertise. This cluster includes the respondent's doctor, pharmacist, a national organization like the American Medical Association, an NIH scientist, the Centers for Disease Control, and the World Health Organization. Our 2020 surveys found that trust in expertise was a strong predictor of an intention to obtain a Covid‐19 vaccination. The *γ* for this relationship was 0.43, indicating that 43% of the variation in vaccination attitude can be accounted for the level of trust in experts. The parallel nature of trust in broadcast news and trust in expertise suggests that many respondents see broadcast news as reflecting the views of relevant experts.^35^ use a similar set of trust questions and characterize the rejection of expertise as anti‐intellectualism. We recognize the long history of anti‐intellectualism in American life and politics^36^ and concur that the rejection of expertise is motivated in part by anti‐intellectualism, but believe that other factors contribute to aggregate trust assessments. In this analysis, we will utilize the broader construct of trust rather than the narrower label of anti‐intellectualism.

Third, we examine the level of trust in Covid‐19 information obtained from online social media sources. This group included measures of trust in Covid information from Wikipedia, WebMD, podcasts, Facebook posts, and YouTube videos. Individuals with a high level of trust in these online sources were slightly more likely to indicate an intention to be vaccinated for Covid‐19 than adults with lower levels of trust in these sources (see Table 5). The *γ* for this relationship was 0.15 – markedly lower than the gammas for broadcast news or local and national experts.^34^ found social media users to be less receptive to expert advice and more hesitant about taking the COVID‐19 vaccine.

Finally, we constructed a scale to measure information acquisition about Covid‐19. Our 2020 survey asked each respondent to estimate the number of times that he or she had attempted to obtain information about Covid‐19 from each of 13 separate sources (see Appendix A for a copy of the questionnaire items used in this analysis). The final scale ranged from 0 to 365. For analysis and presentation purposes, we collapse this distribution into five ordinal categories (see Table 5). The results indicate a positive, but modest, relationship, with a *γ* of 0.18.

### 3.6 The relative influence of these factors on vaccination intention

The preceding discussion identified a number of factors that appear to predict an individual's attitude toward taking the Covid‐19 vaccine. In the course of life, individuals embody all of these characteristics and they interact on a continuing basis. We know, however, that some variables proceed others chronologically or logically. An individual's level of educational attainment may be influenced by age, gender, or race, but the level of education cannot change any of those characteristics. Knowing the chronological and logical order of these independent variables can be valuable for analyzing and measuring the relative influence of each variable on our outcome of interest – each individual's attitude toward taking a vaccination for Covid‐19.

**FIGURE 1:**
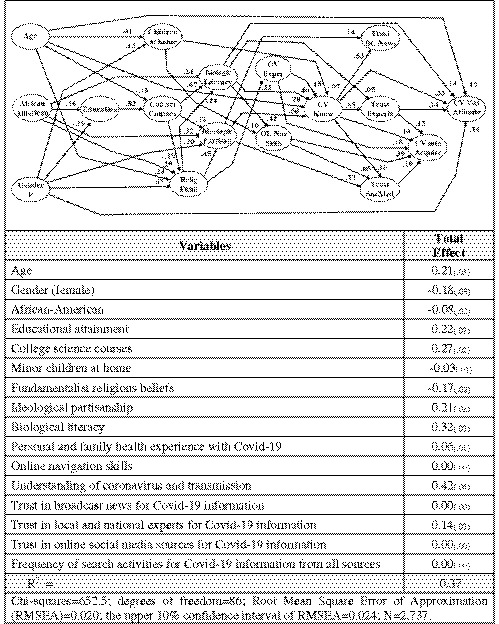
A model to predict attitude toward Covid‐19 vaccination, 2020

For this purpose, we construct a structural equation model (SEM) using LISREL.^37^, ^38^ A diagram of the model is shown in Figure 1, which shows the temporal or causal order of the variables. In the preceding sections of this analysis, we have described each of the variables in the model and the bivariate relationship of that variable to attitude toward Covid vaccination. It is useful to turn now to a discussion of the structure of the model and the model's estimate of the predictive power of each of the independent variables.

The variables are arranged from left to right in an approximate chronological or logical order and influence is presumed to flow from left to right. When two or more variables occur or operate simultaneously, they are placed in the same column, giving them a common left to right location. If one variable is correlated or associated with another variable at a significant level, the two variables are connected with by a path. If two variables are not significantly correlated or associated, the two variables are not connected by a path. Each path has an arrow head on one end, indicating the direction that influence is presumed to flow.

The strength of the relationship between two related variables is indicated by a path coefficient. Path coefficients vary from −1.0 to +1.0 and may be thought of as a partial correlation coefficient. These path coefficients are interesting and numerous in a large model, but we want to know analytically how much total influence each independent variable has on our outcome variable. To estimate the total effect of each variable in the model on the outcome variable, we can multiply all of the coefficients in each pathway from a given independent variable to our outcome variable and sum all of the paths that connect the two variables.

An example may be helpful. If we want to estimate the total effect of coronavirus understanding on vaccination attitude, we can calculate the total effect attributable to the path from CVKnow to TrustExperts to CVVaxAttitude (0.65 × 0.14 = 0.091). There is a second direct (or residual) path from CVKnow to CVVaxAttitude (0.33). This residual path reflects the amount of adjustment needed to reflect the known relationship between CVKnow and CVVaxAttitude. The sum of the two paths is 0.42, which is the total effect shown in the table of total effects in Figure 1.

For analytic purposes, we want to know the total effects (TE) of each of the independent variables in the model. An examination of the total effects table indicates that coronavirus understanding is the strongest single predictor of vaccination attitude (TE = 0.42). Biological literacy is the second strongest predictor (TE = 0.32). Exposure to college science courses (TE = 0.27), the level of educational attainment (TE = 0.22), respondent age (TE = −0.21), and ideological partisanship (TE = 0.21) are moderately strong predictors of a positive attitude toward taking a Covid‐19 vaccine. Religious fundamentalism (TE = −0.17) and female gender (TE = −0.18) had a negative influence on attitude toward Covid‐19 vaccination. It appears that education, biological literacy, and coronavirus understanding are the primary drivers of a positive attitude toward Covid‐19 vaccination.

The model provides some useful insights into the relationship between the several independent variables in our model. Although the bivariate relationship between the amount of Covid‐related information sought and acquired displayed a modest positive relationship (*γ* = 0.18), our SEM indicates that the frequency of Covid‐19 information acquisition did not have a significant marginal influence on vaccination attitudes once the preceding independent variables were taken into account. Similarly, neither the level of trust in broadcast news nor the level of trust in online and social media sources has a significant marginal influence of vaccination attitude once the preceding independent variables are taken into account. The level of trust in expertise did have a marginal positive influence on vaccination attitude (TE = 0.14), indicating that trust in experts was more than a reflection of age, education, biological literacy, and other variables formed earlier in life.

The total model is a good fit for the data (see fit statistics at the bottom on the total effects table in Figure 1) and accounts for 37% of the total covariance in the model.

## 4 DISCUSSION

The formation of an individual's attitude toward taking the Covid‐19 vaccine is a complex process that reflects a combination of early life education and experiences and contemporary political and religious beliefs. Our model points to the central importance of education, biological literacy, and coronavirus understanding in the development of a vaccination attitude. Understanding the science and the related scientific and technological issues does make a difference. To paraphrase Hochschild and Einstein's recent book,^39^ facts do matter for a significant number of American adults.

In an era when terms like “alternative facts” and “fake news” have become commonplace, it is encouraging to see empirical support for the importance of education, biological literacy, and coronavirus understanding on an issue that has been politicized by some of the highest elected officials in the United States. Our data show that the politicization of the coronavirus pandemic has influenced many American adults, but that a significant majority have looked for scientific information from traditional and electronic sources and have been able to use their background biological literacy and current coronavirus information to agree to be vaccinated against the coronavirus. Trust in scientific and health expertise added a marginal total effect of 0.14 in the prediction of vaccination intent, indicating that acceptance of expertise enhanced prior effects of education, biological literacy, and understanding of the coronavirus. In contrast, trust in broadcast information sources and social media sources accounted for no marginal influence on vaccination intent. The absence of a marginal total effect suggests that prior life experience – education, biological literacy, and trust in expertise – fully accounted for individual vaccination intent. This pattern suggests that prior life experiences may have influenced the choice of information acquisition sources.

Our model provides useful insights into the widely observed differential in vaccine hesitancy by race.^7^ The total effect of race – taking into account the full set of life course factors – is −0.08 (see Figure 1). This is a smaller total effect than the raw vaccination record would suggest, but it demonstrates the effect of decades of systemic educational, occupational, and social disadvantage. African‐American respondents were disadvantaged in educational attainment (TE = −0.16), college science courses (TE = −0.13), and biological literacy (TE = −0.38). African‐American respondents were more likely to hold fundamentalist religious beliefs (TE = 0.22). Given this combination of life course experiences, the net marginal impact of being African‐American produced a total effect of −0.08.

Our model demonstrates the structure of religious fundamentalism and Conservative Republicanism on scientific issues like the Covid‐19 pandemic. Our model places the development of religious beliefs prior to the adopting of contemporary ideological partisanship. This means that individuals are more likely to develop religious beliefs and values during their pre‐adult years, but model demonstrates the influence of intervening life experiences. Exposure to college science courses is negatively associated with religious fundamentalism among adults, suggesting that the experiences associated with higher educational attainment and college science courses (TE = −0.19). Religious fundamentalism in strongly related to identification as a Conservative Republican (TE = 0.45). Although the structure of these relationships is complex, it is a good empirical description of the current structure of these factors.

Looking at the full landscape of relevant factors over the life course, our model describes a complex network of life course influences that predict individual intent to take the Covid‐19 vaccine. Education and biological literacy are important baseline influences and will become even stronger influences in the decades ahead. The uniquely American requirement of a full year of science courses during a baccalaureate program in the United States provides a critically important exposure to college science courses, which fosters both biological literacy during the adult years and provides the tools to acquire and utilize current information from traditional media and online sources – more often websites than social media – to enhance an individual's understanding of an emerging public policy issue like the coronavirus pandemic. It is not a simple system, but it appears to work reasonably well.

These results should be encouraging to the scientific community. Apart from our research, many of us engage in extensive efforts to improve the biological literacy of undergraduates and graduate students. Some scientists engage in public outreach efforts formally or informally. Our finding that a substantial proportion of American adults value expertise and utilize it in making personal and public policy judgments indicates that these efforts have produced useful changes in American society and politics. The importance of coronavirus understanding in making a vaccination decision is even stronger evidence of the short‐term influence of expertise. Few American adults would have heard of a coronavirus as an undergraduate and it was almost non‐existent in media prior to the onset of the pandemic. The acquisition of information about the coronavirus and Covid‐19 had to occur in the year prior to our interviews and surveys, indicating that respondents with a high score on the Index of Coronavirus Understanding would have had to acquire almost all of that information and make sense of it with the first year of the pandemic. This kind of learning would not be possible with some prior experience in college science courses – especially biological science courses – and some level of prior interest in understanding science and technology. Collectively, these results indicate that our substantial and continuing investment in scientific and biological education pays dividends in critical situations.

## AUTHOR CONTRIBUTIONS

Jon D. Miller is Director of the International Center for the Advancement of Scientific Literacy in the Institute for Social Research at the University of Michigan and is a Research Scientist Emeritus. He is a political scientist who has studied the development and use of scientific literacy because of its critical role in the functioning of democratic societies in the 21st century. He is a fellow of the American Association for the Advancement of Science. Email: jondmiller@umich.edu. Mark S. Ackerman is a Professor jointly appointed in the School of Information and the Department of Electrical Engineering and Computer Science in the College of Engineering at the University of Michigan, Ann Arbor. He is also the GH Mead Collegiate Professor of Human‐Computer Interaction. Mark is a member of the CHI Academy (HCI fellow) and an ACM Fellow. He publishes widely in collaborative information access, misinformation, and health information from a socio‐technical perspective. Email: ackerm@umich.edu. Belén Laspra Pérez is Assistant Professor of Logic and Philosophy of Science at the Department of Philosophy at the University of Oviedo (Spain). Her current research interests are in scientific literacy, culture of science, and public understanding of science. Email: lasprabelen@uniovi.es. Carmelo Polino is an Assistant Professor in the Department of Philosophy at the University of Oviedo (Spain), and a researcher at Centro Redes (Buenos Aires, Argentina). He holds a PhD in Social Studies of Science, and his main research interests are public understanding of science, STS studies, philosophy of science, and sociology of science communication. Email: polinocarmelo@uniovi.es. Jordan Huffaker is a PhD student in Computer Science and Engineering at the University of Michigan, Ann Arbor. His area of research is social computing. Email: jhuffak@umich.edu.

## DISCLOSURES

The authors report that there are no competing interests to declare.

## Data Availability

The data have been submitted to the Inter‐university Consortium for Political and Social Research (ICPSR) and are being processed. Due to the requirement for extensive remote work during the pandemic, the ICPSR is running behind its normal schedule and estimates that the full data file will be available for secondary analysis by the fall of 2020.

## ENDNOTES

^a^
In this analysis, we will report *gamma* for all of the relationships between two ordinal variables.^8^ examined measures of the relationships between non‐interval variables and identified gamma as a proportional‐reduction‐of‐error (PRE) measure for use with two ordinal variables. A dichotomous variable may be treated as either an interval variable or an ordinal variable.

^b^
The five items used are: (1) agreement that “There is a personal God that hears the prayers of individuals,” (2) agreement that “The Bible is the actual word of God and is to be taken literally,” (3) the self‐reported number of times that each respondent attends a religious service in a typical week, (4) the self‐reported number of times that each respondent prays during a typical week, and (5) disagreement that “Human beings, as we know them today, developed from earlier species of animals.”

## APPENDIX A

### Items used in computing an index of Covid‐19 information acquisition

The following battery of questions about seeking information about Covid‐19 were used to construct an Index of Covid Information Acquisition that ranges from 0 to 365.

Q11. Thinking about information about Covid‐19, how many times have you done each of the following activities during 2020? If you have not done an activity, please enter 0 and go to the next item.

**Table:**

	Number of times
Talked to a doctor about Covid‐19.	A
Looked for information about Covid‐19 in a public library in person or online.	B
Read a newspaper or magazine article about Covid‐19.	C
Watched a television (network or cable) news story about Covid‐19.	D
Looked for information about Covid‐19 on the Internet.	E
Talked to other members of my family about Covid‐19.	F
Prayed for relief on this matter.	G
Talked to friends or co‐workers about Covid‐19.	H
Read a story on my Facebook newsfeed about Covid‐19.	I
Read about Covid‐19 on Twitter, Reddit, or similar social media sites.	J
Watched a video or news story about Covid‐19 on YouTube or similar sites.	K
Listened to a podcast about Covid‐19.	L
Read or contributed to a blog about Covid‐19.	M
Printed or saved an article from the Internet about Covid‐19.	N
